# Assisted Reproductive Technology and Anorectal Malformation: A Single-Center Experience

**DOI:** 10.3389/fped.2021.705385

**Published:** 2021-09-16

**Authors:** Chiara Iacusso, Barbara Daniela Iacobelli, Francesco Morini, Giorgia Totonelli, Milena Viggiano, Leonardo Caforio, Pietro Bagolan

**Affiliations:** ^1^Neonatal Surgery Unit, Department of Neonatology, Bambino Gesù Children's Hospital, IRCCS, Rome, Italy; ^2^Obstetrics and Gynecology, Department of Neonatology, Bambino Gesù Children's Hospital, IRCCS, Rome, Italy; ^3^Department of Systems Medicine, University of Rome “Tor Vergata”, Rome, Italy

**Keywords:** anorectal malformation, assisted reproductive techniques, VACTERL, congenital anomalies, malformation anomalies

## Abstract

**Background:** Assisted reproductive technologies (ART) are becoming widespread, accounting for approximately 2% of all births in the western countries. Concerns exist on the potential association of ART with congenital anomalies. Few studies have addressed if a relationship exists between ART and the development of anorectal malformation (ARM). Our aim was to analyze the relationship between ARM and ART.

**Methods:** Single-center retrospective case control study of all patients treated for ARM between 2010 and 2017. Patients with bronchiolitis treated since 2014 were used as controls. Variables analyzed include the following: prevalence of ART, gestational age, birth weight, and maternal age. Patients with ARM born after ART were also compared with those naturally conceived for disease complexity. Fisher's exact and Mann-Whitney *U*-tests were used as appropriate.

**Results:** Three hundred sixty-nine patients were analyzed (143 cases and 226 controls). Prevalence of ART was significantly higher in ARM patients than in controls [7.6 vs. 3.0%; odds ratio: 2.59 (95% CI, 0.98–0.68); *p* = 0.05]. Among ARM patients, incidence of VACTERL association (17%) is more frequent in ART babies.

**Conclusion:** Patients with ARM were more likely to be conceived following ART as compared with controls without congenital anomalies. Disease complexity of patients with ARM born after ART seems greater that those born after nonassisted conception.

## Introduction

In Western countries, infertility affects 10–15% of couples of reproductive ages worldwide, and this scenario leads to progressive increase in the use of assisted reproductive techniques (ART), such as *in vitro* fertilization (IVF), intracitoplasmatic sperm injection (ICSI), and others (1). Since 1978, when the first child, Louise Brown, was conceived after IVF, ART is gaining ground. In the USA, the number of infants conceived after ART increased from 21,943 in 1997 to 66,706 in 2013, with more than 1.5% of total births in 2013 (2, 3). Similar trend is observed in Europe, where the infants born after ART increased from 35,314 in 1997 (18-country Europe) to 143,844 in 2012 (34-country Europe) (1). Due to the widespread of ART, these techniques account for approximately 2% of all births in the European countries (4) with a considerable disparity in live birth rates and outcome between regions. As a consequence, it is estimated that over 200,000 babies worldwide are annually born after ART (5, 6). However, possible maternal and fetal adverse outcome are source of debate. Actually, concerns exist on the potential association of ART with congenital anomalies. While most children born after ART are healthy, previous studies also reported on health effects, such as higher frequencies of prematurity and low birth weights (7). Several studies also reported an increased risk of major congenital malformation following ART ranging from 29 to 41% (8–12). Few have addressed if a relationship exists between ART and the development of anorectal malformation (ARM) (12). ARM represents a rare birth defect of the anus and rectum with largely unknown causes. Approximately one in 2,500–3,500 babies are affected worldwide.

The aim of the present study was to analyze the potential relationship between a major congenital anomaly, as ARM, and ART. Furthermore, we compared ARM patients conceived after ART vs. ARM patients naturally conceived, to highlight any difference on its prevalence and severity between the two groups.

## Materials and Methods

This is a retrospective case control study of all patients born between 2010 and 2017 and admitted for ARM to our hospital. Personal interviews were conducted by a surgeon with parents of affected children within the first admission after childbirth. For each patient with ARM (case), 1.6 controls were included. Control group was represented by infants admitted for bronchiolitis at our medical department of neonatology during the same period. The same family interview was conducted by a neonatologist to parents of affected children. Exclusion criteria for the control group were the association with a major congenital anomaly, very low birth weight, and severe prematurity. We analyzed the following variables: prevalence of ART, gestational age, birth weight, and maternal age. In patients born after ART, we did not differentiate between IVF and ICSI. Furthermore, patients with ARM born after ART were also compared with those with ARM born after nonassisted conception for disease complexity (association to vertebral, anal, cardiovascular, tracheoesophageal, renal, limb (VACTERL) or associations to genetic disorders). The *t*-test was used to assess difference in maternal age at birth between ARM cases and controls. For calculations of other differences, nonparametrical measurement methods were used (Mann-Whitney *U*-test, Fisher's exact test). In case of a normal distribution, the mean value was calculated, otherwise the median. Software GraphPad Prism 5.0 Macintosh Version (GraphPad Software, San Diego, California, USA, http://www.graphpad.com). Two-tailed *p* < 0.05 was considered statistically significant. Results are prevalence, odds ratio (OR), and median (95% interquartile range).

## Results

In an 8-year period, 369 patients were analyzed: 143 consecutive cases of ARM and 226 controls. Prevalence of ART was significantly higher in ARM patients than in controls [7.6 vs. 3.0%; OR: 2.59 (95% CI, 0.98–0.68); *p* = 0.05]. The birth weight of ARM patients was lower as compared with control group (*p* = 0.001). No significant difference was detected between the two groups in terms of gestational age and maternal age. Table 1 shows the main results. Analyzing the complexity of ARM patients born after ART or naturally conceived shows that prevalence of VACTERL association was significantly higher in patients with ARM born after ART [45.5 vs. 19.7%; OR: 3.39 (95% CI, 0.96–0.12); *p* = 0.04] (Table 2).

**Table 1:** Comparison between anorectal malformation (ARM) population and controls.

	**ARM**	**Controls**	***p*-value**
Number of pts	143	226	
ART	11 (7.6%)	7 (3.0%)	0.04
Gestational age (weeks)	38 (27–42)	39 (37–42)	ns
Birth weight (g)	2,865 (710–4,600)	3,210 (1,890–4,500)	0.001
Maternal age (years)	32 (20–48)	33 (19–48)	ns

**Table 2:** Comparison between patients with anorectal malformation born after assisted reproduction technology (ART) or naturally conceived.

	**Born after ART**	**Naturally conceived**	***p*-value**
Number of pts	11	132	
Gestational age (weeks)	38 (30–38)	38 (27–42)	ns
Birth weight (g)	2,300 (1,250–3,920)	2,880 (710–4,600)	ns
Maternal age (years)	36 (20–48)	32 (20–43)	ns
VATER/VACTERL	5 (45.5%)	26 (19.7%)	0.04
Genetic disorders	1 (9%)	9 (6.8%)	ns
Death	1 (9%)	5 (3.7%)	ns

## Discussion

In the present study, we observed a significant increased prevalence of ART in our ARM population as compared with the control group, suggesting an association between ART and ARM development. Moreover, patients with ARM+ART showed a more complex disease as suggested by a higher prevalence of VACTERL association. These data suggest that ART may be associated with a more profound disruption in embryogenesis, thus leading to the development of a wider spectrum congenital abnormality such as VACTERL association.

Scant data exist so far specifically on the potential association of ART with the development of ARM (9), that is a rare malformation of the lower digestive tract, currently not associated to specific risk factors (13). The congenital anomalies most studied in terms of association with ART are cardiac abnormalities. Children born after ICSI showed variable birth outcomes, from threefold increased risk of congenital heart disease, a twofold risk of major congenital cardiac defects (aortic stenosis, wide atrial septal defect, coarctation of the aorta, Ebstein anomaly, tetralogy of Fallot, truncus arteriosus), and 50% increased risk of minor cardiac defects (small septal defects, patent ductus arteriosus, patent foramen ovale) to no differences (14–16). Data from a recent meta-analysis including 57 cohort studies and involving 120,000 ART infants and more than a million naturally conceived infants aimed to provide whether the relationship between ART and congenital anomalies exists or not. In addition to an increased risk of some adverse outcome in ART pregnancies, they found a 33% increased risk of congenital anomalies as compared with those spontaneously conceived (17). Despite several issues complicating the interpretation of available data, a recent large registry-based study comparing the prevalence of birth defect in ART and non-ART children reveals a significant higher relative risk for nonchromosomal birth defects (e.g., EA/TEF, ARM, lower limb deformity) with ART use (18). The manipulation of the gamete or the embryo and alterations of the environment where the gametes normally grow may increase the risk of abnormal development (19), thereby increasing the risk of congenital anomalies. Focusing on ARM development in ART pregnancies, the manipulation of the embryo before implantation may affect the cloacal and hindgut development, thereby leading to its abnormal anatomy. Classically, the anorectal canal results from a “late shift” of the rectum (“caudal migration”) or a shift of the caudal cloaca to the tail groove (20). Recently, Kluth and colleague demonstrated that the area of the future anal orifice is formed in an early phase of embryo development (first 2–4 weeks) and forms a fixed point in cloacal and hindgut development. Using scanning electron microscopy (SEM), they showed that early abnormalities of the dorsal caudal membrane and the dorsal cloaca, causing their absence, might be associated to an abnormal development of both the anal orifice and the lower rectum (21).

Several studies suggest an increased risk of major congenital malformations following ART, ranging from 29 to 41% (5). Even prior to the pioneering efforts of Steptoe and Edwards (2), concern had been raised over the risk that infants born after ART would be affected by congenital abnormalities. The initial studies showed no significant difference in terms of congenital anomalies when comparing ART infants with the general population (22–24). In the early 2000s, some studies raised doubts on these reassuring results showing increased risks of specifically associated anomalies in pregnancies after ART, and meta-analyses lead to similar conclusions. Rimm et al. found an OR for associated anomalies of 1.29 (95% CI: 1.01–1.67) (25) and Wen et al. an OR of 1.37 (95% CI: 1.26–1.48) (26). From these data, associated anomalies may be expected to be 25–70% more prevalent in ART pregnancies as compared with children naturally conceived. Later, Hansen and colleagues tried to quantify the risk of birth defects in ART infants compared with non-ART infants overall. They published a systematic review and meta-analysis including 45 selected papers and 92,671 ART infants. They found a 30% increased risk of birth defects in children born after ART with an OR of 1.32 (95% CI: 1.24–1.42), meaning that for a population with a background birth defects of 5%, this equates to an absolute risk of almost 7% (27, 28). However, these meta-analyses included a very long-time period in a field that is moving fast. Technologies and laboratory conditions evolve very rapidly over time, and these changes strongly impact on ART results. Our series confirms an association between ART and the risk of developing congenital anomalies, in a recent series, collected over a relatively short time, that may attenuate the impact of technological advances on the outcomes of ART.

The present study has some limitations. First, the limited number of patients did not allow to differentiate between the types of ART procedure (ICSI vs. IVF). Additionally, the data were based on maternal report, opening to potential misreport biases. We were not able to differentiate between the risks related to the procedures themselves and that related to the couple subfertility/infertility. Singleton pregnancies achieved by assisted reproduction seem at higher risk than spontaneous pregnancies for adverse perinatal outcomes (26, 29). However, in Allen et al. (29) reviewing the effect of ART on perinatal outcomes, provided guidelines to optimize obstetrical management and counseling of Canadian women using ART. The conclusion was that spontaneous pregnancies in untreated infertile women might be at higher risk for obstetrical complications and perinatal mortality than spontaneous pregnancies in fertile women, independent of ART. Also, pregnancies achieved by ovarian stimulation with gonadotropins and intrauterine insemination are at higher risk for perinatal complications. Therefore, subfertility/infertility itself may contribute to the increased risk of adverse perinatal outcome (13). Other factors that may affect the neonatal outcome include parental factors and behavior, IVF vs. ICSI, type and dose of medications, gamete/embryo culture media, frozen vs. fresh embryo, and others.

## Conclusion

In conclusion, our data show a higher incidence of medical conception in children affected by ARM than in the general population, suggesting a potential relationship between ART and ARM development. Furthermore, ARM born after ART seem to present a higher complexity as compared with naturally conceived ARM. Future parents should be informed about the potential increased risk of birth defects determined by subfertility and possibly, by ART exposure, and ART pregnancies should be followed with particular attention to potential congenital structural abnormalities. Further prospective studies, including data from the National Database of ART, are needed to confirm our data and clarify the connections, if any, between ART and major congenital anomalies.

## Data Availability Statement

The raw data supporting the conclusions of this article will be made available by the authors, without undue reservation.

## Author Contributions

All authors listed have made a substantial, direct and intellectual contribution to the work, and approved it for publication.

## Conflict of Interest

The authors declare that the research was conducted in the absence of any commercial or financial relationships that could be construed as a potential conflict of interest.

## Publisher's Note

All claims expressed in this article are solely those of the authors and do not necessarily represent those of their affiliated organizations, or those of the publisher, the editors and the reviewers. Any product that may be evaluated in this article, or claim that may be made by its manufacturer, is not guaranteed or endorsed by the publisher.
